# Mechanical Behavior—Microstructure Relationships in Injection-Molded Polyamide 66

**DOI:** 10.3390/polym10101047

**Published:** 2018-09-20

**Authors:** Noëlle Billon, Joan Giraudeau, Jean Luc Bouvard, Gilles Robert

**Affiliations:** 1 MINES ParisTech, PSL Research University, CEMEF, CNRS UMR 7635, CS 10207, CEDEX, 06904 Sophia Antipolis, France; joan.giraudeau@mines-paristech.fr (J.G.); jean-luc.bouvard@mines-paristech.fr (J.L.B.); 2 Solvay Engineering Plastics, Technyl Innovation Center, Avenue Ramboz—BP64, 69192 Saint Fons, France; gilles.robert@solvay.com

**Keywords:** microstructure, mechanical properties, injection molding, polyamide 66

## Abstract

Clear relationships between the semi-crystalline microstructure of injection molding polymers and their mechanical behavior are not yet totally established for all polymers. Part of this relative lack of understanding is because an unambiguous scientific approach is difficult to build up. The processing of samples promotes a microstructure which is not uniform and can be described in various ways on different scales. This introduces uncertainty in the correlations. Most completed studies were conducted on polyolefin, which exhibits an evolution of microstructure that is quite easy to observe and to correlate to mechanical properties. This paper intends to illustrate a more diffuse case. To achieve this, combined characterizations along the flow path and throughout the thickness of a plaque as well as characterizations of the local microstructure and tensile behavior of polyamide 66 are described. The microstructure was explored in terms of skin-core structure, spherulites sizes, crystallinity ratio and lamellae organization. Mechanical properties were addressed with non-monotonic tests with the use of DIC (Digital Image Correlation) to assess true behavior. The effect of humidity is also accounted for. It is demonstrated that small changes in lamellae or interlamellar amorphous phase are likely to be responsible for non-uniform mechanical properties, whereas more macroscopic levels (skin core structure, spherulites level of crystallinity ratio) appeared to be irrelevant levels of description. Consequently, the usual simplified analyses based on optical microscopy and differential scanning calorimetry (DSC) can be inefficient in improving knowledge in that field.

## 1. Introduction

Injection molding is a common process for manufacturing thermoplastic parts or samples. However, it induces a heterogeneous microstructure that responds to heterogeneous thermo mechanical histories during the processing itself.

From a technological point of view, injection molding involves three main steps: filling, packing-holding and cooling. Firstly, the molten thermoplastic is forced into the mold. The contact between the hot polymer and the cold mold induces an important thermal gradient throughout the thickness as well as a restriction of the molten flow “channel”. Complex flow fields of the molten polymer then promote elongation or shear stress (strain) depending on the location and time. Once the mold is filled, pressure is applied to counterbalance shrinkage during the cooling. Then a pressure gradient is built up along the flow direction. Finally, the part is cooled down and ejected. Consequently, local thermo-mechanical environments during the formation are piloted by global conditions: part/mold geometry, molten material properties, crystallization ability and machine set-up. The specific profiles of the pressure, temperature, strain rate and stress can be estimated by computational simulations and, in some cases, assessed by adequate mold instrumentation. This has led to numerous studies in the past (for example [1]) but general features of the relationships between processing conditions and properties are still an open question [2].

Any of these thermo-mechanical evolutions constrain variations of crystallization conditions and impact the crystalline morphology as well as the crystalline content [3]. In particular, cooling rates and flow conditions, by affecting the nucleation and the growth of crystalline entities, lead to a laminate organization known as the ‘skin-core’ structure [4,5]. At low optical resolution, three main layers are often named:

- the outer skin layers presenting a high degree of molecular orientation, shish-kebab like structures and lower crystallinity ratio [5,6];
- an intermediate zone where the combination of shear flow and cooling rate forms a microstructure composed of deformed spherulites or comet-like entities [7,8];
- a core zone, assumed to crystallize under static conditions with almost negligible orientation and a spherulitic type of crystallization eventually affected by pressure [9].

This skin-core structure and the crystallinity ratio are the scales on which microstructure is often studied, without proof that these scales are the only relevant ones that should be taken into consideration (see for example [2]).

However, microstructure can be described (and can vary) in a much richer manner on several scales including crystallographic system and perfection, lamellae thickness, spherulites organization or more complex macroscopic heterogeneity throughout and along the part [10].

The mechanical properties of polymer parts are controlled by the microstructure [11,12,13,14,15], in a manner not completely understood. Additionally, moisture may need to be considered [16,17,18].

The goal of this general study is to contribute to the understanding and the modeling of the relationships between the processing and the mechanical behavior of injection-molded polymers. In this framework, a polyamide 66 is considered as an example.

Starting from the common-sense conclusion that straightforward relationships between processing conditions and mechanical behavior cannot be the most general route, mechanical characterization should be performed on samples reproducing (or exhibiting) a relevant and controlled microstructure. Then mechanical characterization should deal with the exploration of the relationships between the microstructure and the induced properties.

First, the level of description of the microstructure to be accounted for must be defined and its range of variation should be explored. The present paper deals with this particular step. It offers a first and as complete as possible characterization of the local microstructure and the local mechanical behavior of a polyamide 66. As the targeted application is injection molding, we focus on injection-molded plaques. The analyses combine optical, thermal, and X-ray diffraction measurements as well as tensile tests. From these observations, the most relevant level of microstructure description is proposed for further and broader study.

## 2. Materials and Methods

### 2.1. Materials

The material was a polyamide 66 (PA 66) of average molar mass 16,000 g mol^−1^ supplied by Solvay^®^ (St. Fons, France). The material was molded, after drying, into a 3.2-mm thick plaque. Injection conditions were named “standard”. The plaques were rectangular with dimensions of 100 × 300 × 3.2 mm^3^. The injection gate was flat along the width of the plaque.

Samples were taken from those plaques carefully to avoid overheating the material without lubrication. They were designed either to involve the entire thickness of the plaques (3.2 mm) or a given part (close to skin or core) in a particular zone of the plaque (e.g., closer or further away from the gate). The thicknesses of the different samples (close to the skin or core) were chosen depending on the requirement of the experimental techniques.

The water content of each sample was estimated by weighing it and comparing it to the weight of the same sample after drying. The drying protocol involved 18 h at 90 °C under vacuum (100 mbar). This does not ensure a true 0 wt % humidity, but the water content should be less than 0.5 wt % in these conditions. The “as-dried” material is referred to as 0 wt % H_2_O in the following.

The microstructure was observed locally using three techniques.

### 2.2. Polarized Light Microscopy

Polarized optical microscopy was used to assess the skin-core and spherulitic levels. Observations were made on 10-µm thick microtomed cuts (with glass blades). The cross-section was chosen to observe the evolution of morphologies, with a thickness of approximately 250 µm. The transversal direction was preferred over the flow direction. Observations were made with a Leica microscope (Paris, France) with 90° polarized light. Measurements and picture treatment were performed by a computer.

### 2.3. Differential Scanning Calorimetry

A thermal analysis was carried out with a Perkin-Elmer DSC 8000 differential scanning calorimeter (Les Ulis, France) to analyze the melting traces. In brief, 3- to 5-mg samples (500 µm thick) were tooled from the surface and core of the plaque, ensuring a good thermal contact with differential scanning calorimeter (DSC) pans (aluminum). Temperature calibration and melting enthalpy were performed with indium and zinc. Each thermogram was obtained from 0 °C to 300 °C at a heating rate of 10 °C/min.

The classical Thomson-Gibbs equation was used to assess the thickness of crystalline lamellae (*L_c_*) from their melting trace (*T_f_*):

(1) $$T_{f}=T_{f}^{0}\left(1-\frac{2\sigma_{e}}{\Delta H_{f}^{0}\cdot \rho_{c}\cdot L_{c}}\right)$$

where *σ_e_* is the basal surface free energy per unit area, *ρ_c_* is the density of the crystal, Δ*H*^0^*_f_* is the melting enthalpy of a perfect crystal (J·kg^−1^) and *T*^0^_*f*_ is the melting temperature of an infinite crystal. For calculations, *σ_e_* = 0.0296 J·m^−2^, *T*^0^_*f*_ = 270 °C and *ρ_c_* = 1240 kg·m^−3^ were used [19].

### 2.4. Wide-Angle X-ray Scattering (WAXS)

For a more in-depth observation of the crystalline phase, WAXS was performed using CuKα radiation (λ = 1.540 Å). The X-ray beam was filtered with Ni at 45 kV and 30 mA and collimated to circular dimensions of 2 mm. We used a horizontal goniometer (4° to 70°). Some circular samples with diameters of Ø = 15 mm and thicknesses of *e* = 500 µm were machined from the plaque. Sampling sites were similar to those chosen for DSC and the exposure time varied from 1 min to 2 min for each sample.

**Figure 1:**
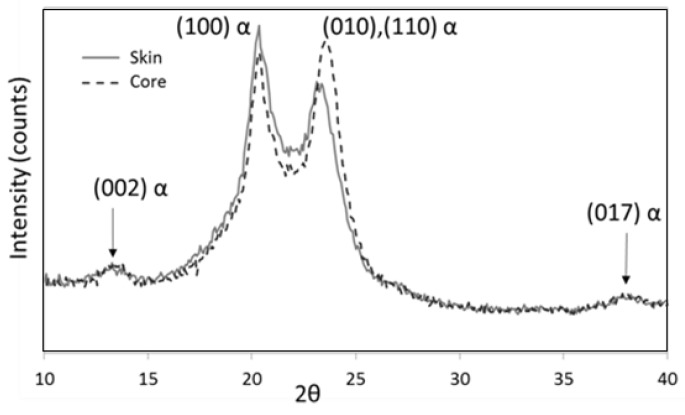
Typical X-ray diffraction for polyamide 66 (PA 66); intensity vs. 2θ (θ is the diffraction angle).

Typical results are depicted in Figure 1. They only revealed α crystalline form [19] with one main reflection associated with the (100) planes and a second combining (010) and (110) reflections.

**Figure 2:**
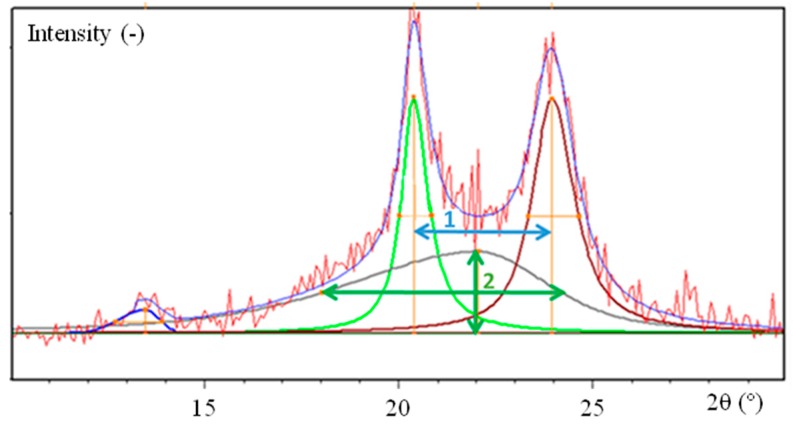
Typical decomposition for diffraction.

A typical decomposition of (002), (100), (010)/(110) and amorphous contributions are depicted in Figure 2. To achieve this analysis, Pearson VII functions were used. The amorphous contribution was extracted according to the literature [20,21] with a maximum contribution close to 2θ of 21°.

The microstructural parameters that could be estimated are:

- the crystalline perfection index, by measuring the angular difference between the greater of the two main reflections [20] (distance 1 in Figure 2);
- the constrained state of the amorphous content, by measuring the half-width of the amorphous halo (distance 2 in Figure 2);
- the crystallinity index, which is the ratio between the sum of the area of crystalline peaks and the total profile area including the amorphous contribution [22];
- the lamellae size (perpendicular to the (100) plane) from the half-width of the (100) peak [23].

Additionally, long periods using SAXS (Small-Angle X-ray Scattering) characterizations were also determined and some two-dimensional (2D) patterns (Debye Scherrer technique) allowed the assessment of crystalline anisotropy in the plane of the plaques.

### 2.5. Dynamic Mechanical Thermal Analysis (DMTA)

DMTA were used as a first estimate of the viscoelastic properties of the material.

Parallelepiped shaped samples (with a length of 30 mm, a width of 5 mm and a thickness of 1 mm) were characterized in tension-tension mode with a Bohlin Treta 2000 DMTA apparatus.

The sample direction was either parallel or perpendicular to the direction of the injection flow.

The temperature ranged between −20 °C and +160 °C with a heating rate of 2 °C/min. The frequency ranged from 0.1 Hz to 10 Hz. A ±5 µm maximum displacement was imposed, which induced a ±10^−3^ maximum strain.

### 2.6. Tensile Tests

Tension was chosen to analyze the behavior of the polymer at a high strain level. Uploading-unloading cycles were used to address viscoelasticity of the latter. Samples were tooled, parallel or perpendicular to flow directions, with a gauge length of 15 mm, a width of 10 mm and thicknesses of 3 mm (for the full thickness samples) or 1 mm (for the skin and core samples).

Specific devices were used to impose a constant true strain rate (4 10^−3^ s^−1^ and 4 10^−2^ s^−1^) in the central zone of the sample during the two steps. Strain rates were chosen to avoid creeping and self-heating. Digital image correlation (DIC) enabled the recording of a 2D strain field on the sample surface, located in the central zone of the sample. Local stress was deduced from the measured force, assuming transverse (throughout the thickness) isotropy.

**Figure 3:**
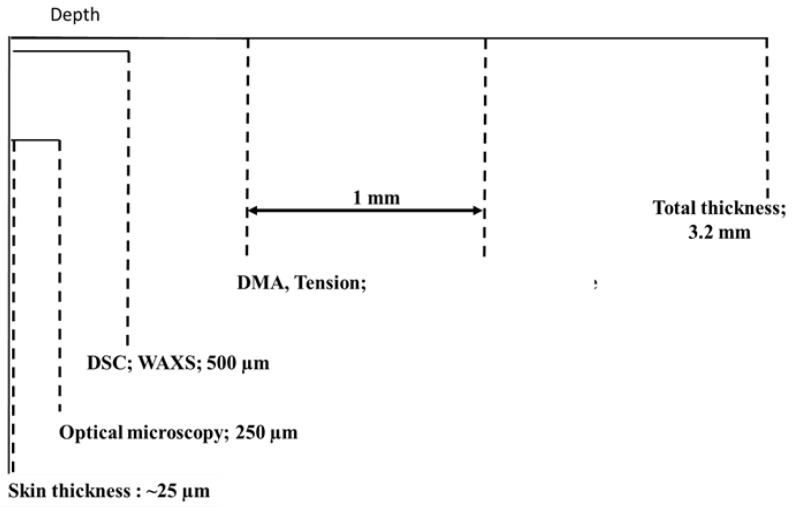
Sampling and characterizations within the thickness.

Figure 3 illustrates the sampling strategy of the study as a function of the different techniques.

## 3. Results

### 3.1. Macroscopic Analysis along the Flow Direction Gradient and the Effect of Humidity

Mechanical testing was performed for specimens machined parallel and perpendicular to the flow direction. We noticed that the processing conditions used in this study did not promote significant crystalline nor mechanical anisotropies. Therefore, we will present only the results obtained parallel to the flow direction.

**Figure 4:**
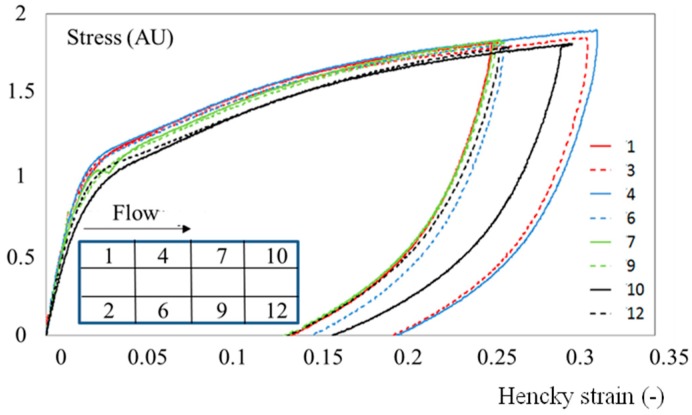
Loading-unloading stress vs. strain cycles under tension at 23 °C. The water content is 2 wt.%. The strain rate is 4 10^−3^ s^−1^. Figures in the legend refer to the location in the plaque as depicted in the schematic on the left.

**Figure 5:**
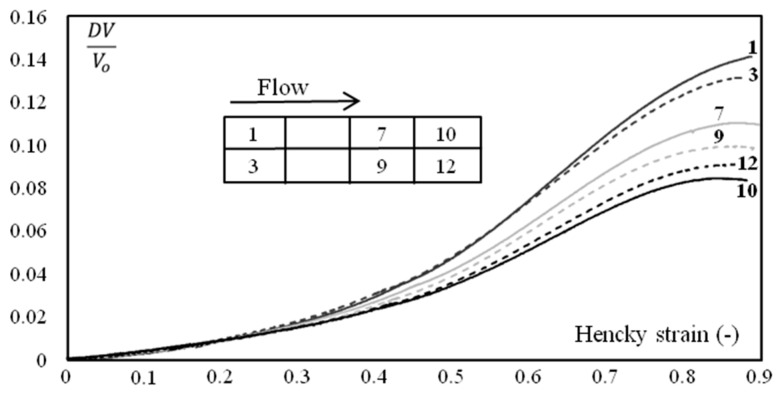
DV/Vo (Equation (2)) as a function of axial strain during monotonic tension conditions at 23 °C. The water content is 7 wt.%. The strain rate is 4 10^−3^ s^−1^. Figures in the legend refer to the location in the plaque as depicted in the schematic on the left.

However, the mechanical behavior of the polymer was not perfectly uniform and depended, to some extent, on the location along the plaque (Figure 4). A quick glance could suggest that this was a kind of scattering. However, a more in-depth analysis showed that there was a coherent evolution (though weak) from the gate to the bottom of the mold (zones 10 and 12 in Figure 4). This trend was more significant when considering the variation of volume during the application of tension (Figure 5):

(2) $$\frac{DV}{V_{0}}=\exp\left(\varepsilon_{1}+2\;\varepsilon_{2}\right)-1$$

where *DV*, *Vo* are the volume variation and the initial volume of a volume element of the sample, respectively. For their part, *ε_1_* and *ε_2_* are the local true axial and local true transversal strains, as measured during the application of tension.

The material was more rigid and the deformation was less isochoric close to the gate. Samples were always extracted at the same location and tested in the same conditions; scattering appeared to be less important than the differences observed in Figure 4.

**Figure 6:**
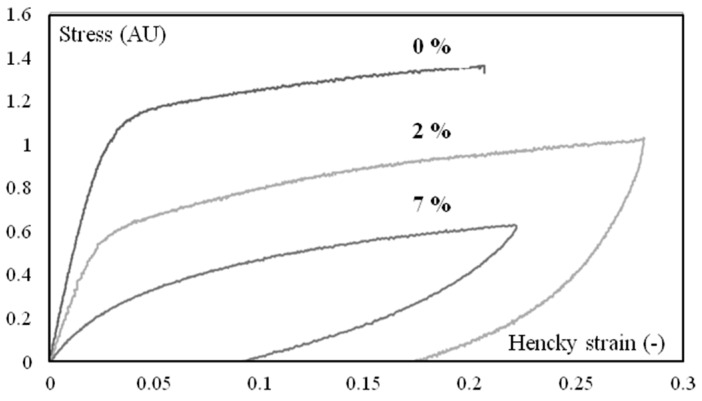
Loading-unloading stress vs. strain cycles under tension at 23 °C for three different water contents. The strain rate is 4 10^−3^ s^−1^. Samples were extracted in the first third of the plaques (close to the gate).

**Figure 7:**
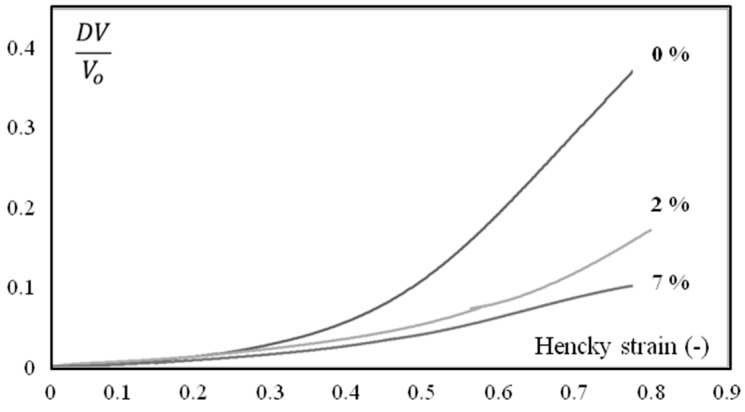
DV/Vo (Equation (2)) as a function of axial strain during monotonic tension conditions at 23 °C for different water contents (in weight). The strain rate is 4 10^−3^ s^−1^. Samples were extracted in the first third of the plaques (close to the gate).

**Figure 8:**
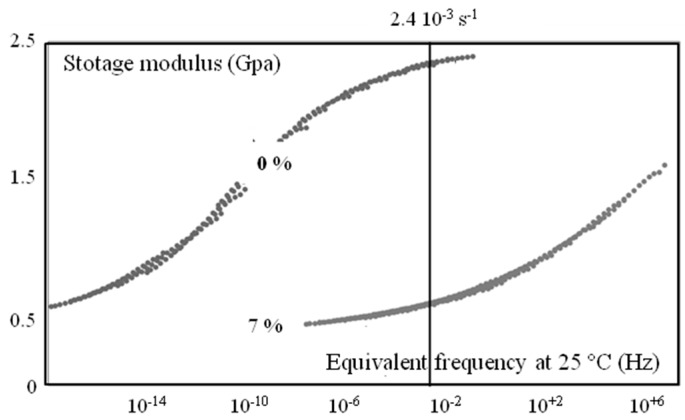
Master curves at a reference temperature of 25 °C for two water contents in the core of the plaque. The straight line illustrates the equivalent stain rate for tensile conditions (23 °C and 4 10^−3^ s^−1^).

Humidity highly influences mechanical behavior (Figure 6 and Figure 7), which could be correlated to a shift of α transition temperature (Figure 8). Indeed, the behavior of the material is more viscoelastic and closer to incompressibility (isochoric) above glass transition. Brittleness increases in the glassy state (0% in Figure 6 and Figure 12).

This evolution can be explained by the equivalence between temperature and humidity that has previously been pointed out (e.g., [24]). On the other hand, observations along the plaques need more information regarding microstructure. That is the goal of the following section.

### 3.2. Morphological Evolutions

**Figure 9:**
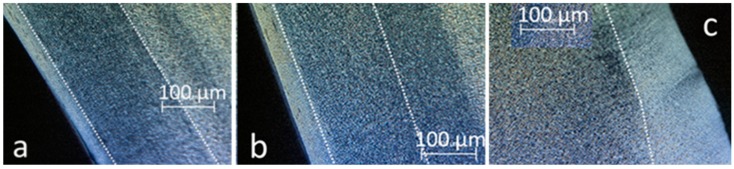
Microscope observations of the plaque: (**a**) 60 mm away from the gate; (**b**) 225 mm away from the gate; (**c**) 275 mm away from the gate.

Figure 9 shows optical micrographs of slices close to the surface. Only a 25-µm thick skin layer could be identified on the extreme surface. This was possible thanks to its optical birefringence, making it sensitive to the relative orientations of the sample and of the polarizers. In that zone, no spherulites could be observed. At a greater depth, spherulites were visible with an increasing average diameter toward the core. Diameters increased from 5 μm to 15 μm. Beyond a depth of 150 µm, this diameter appeared to be more uniform.

**Figure 10:**
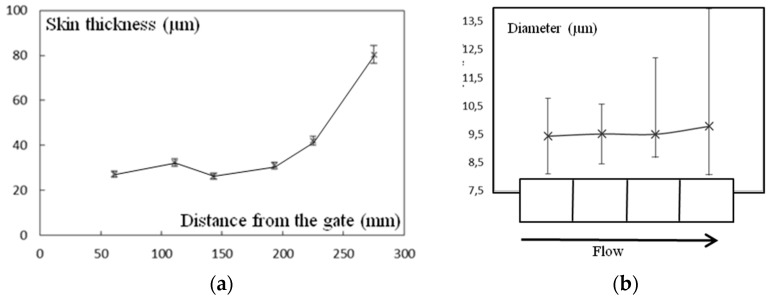
(**a**) Thickness of the skin zone vs. distance from gate; (**b**) diameter of spherulites as a function of their position in the plaque. Bars are scattering bars.

The thickness of skin zone increased from the gate to the bottom of the plaque (Figure 10a). An intermediate gradient was not observed in the latter zone, i.e., three zones can be defined close to the gate and only two close to the bottom of the mold. In the core zone the scattering of spherulite diameters increased, keeping the average value unchanged (Figure 10b). However, those changes appeared to be quite low and it would be difficult to argue that they are responsible for mechanical evolution.

**Figure 11:**
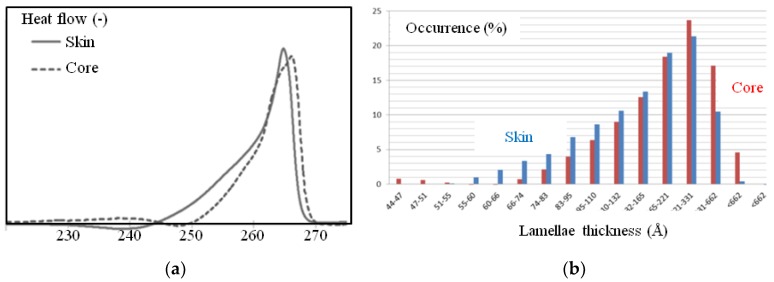
(**a**) Differential scanning calorimetry (DSC) traces of surface and core samples (endotherms are positive values); (**b**) lamellae thickness distribution deduced of (a) and Equation (1) in the core (red) and in the skin (blue).

The DSC measurements provided additional information suggesting that the lamellae structure could be different in the area close to the surface of the plaque compared to that in the core. Figure 11 depicts typical DSC traces during melting for surface and bulk samples. Each endotherm peak presents a well-known (for polyamide 66) shape [8,21,25]. This could (though not exclusively) be explained by a non-uniform thickness of the crystalline lamella. The Thomson-Gibbs equation suggested values from 50 Å to 700 Å with an average thickness, <*L_c_*>, of 240 Å for surface samples and of 331 Å for the core (Table 1), with a higher homogeneity in lamellae thicknesses.

Finally, the apparent crystallinity ratio in mass, Xc, was estimated to be close to 40% for the skin and 38% for the core (Table 1).

X-ray scattering revealed that only the α triclinic crystalline form was present. According to the above analysis, the core could be more crystalline, with thicker lamellae, a longer period and a more perfect crystal than the surface samples. Moreover, the decrease of ∆θ_am_ values could be interpreted as a higher constraint level of the inter-lamellar amorphous phase (Table 2).

It was not possible to draw a more precise description but combining all these observations, one can imagine that the surface exhibited a less “perfect” crystalline organization and a more mobile amorphous phase and that these characteristics become more and more probable along the flow direction.

### 3.3. Mechanical and Viscoelastic Behavior

Linear viscoelastic properties, as revealed by DMTA, did not vary throughout the thickness and along the plaque. The α transition temperature as well as moduli were equivalent in the core, at the surface and all along the plaque.

**Figure 12:**
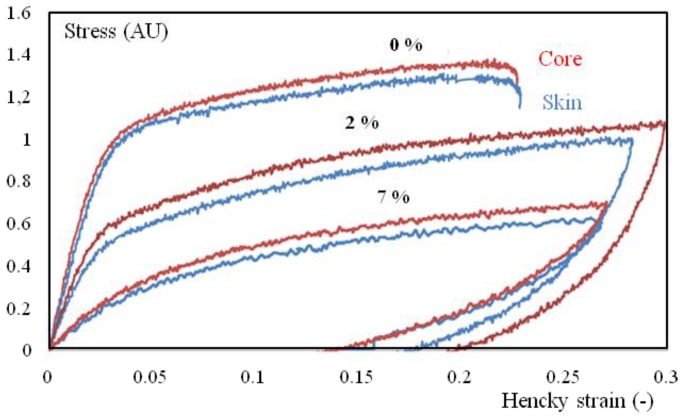
True stress-true strain cycles for tensile tests of core (red), and surface (blue) specimens with different water contents (in wt.% on the curves). The strain rate is 4 10^−3^ s^−1^ and the temperature is 23 °C. Samples were extracted from the first third of the plaque (close to the gate).

**Figure 13:**
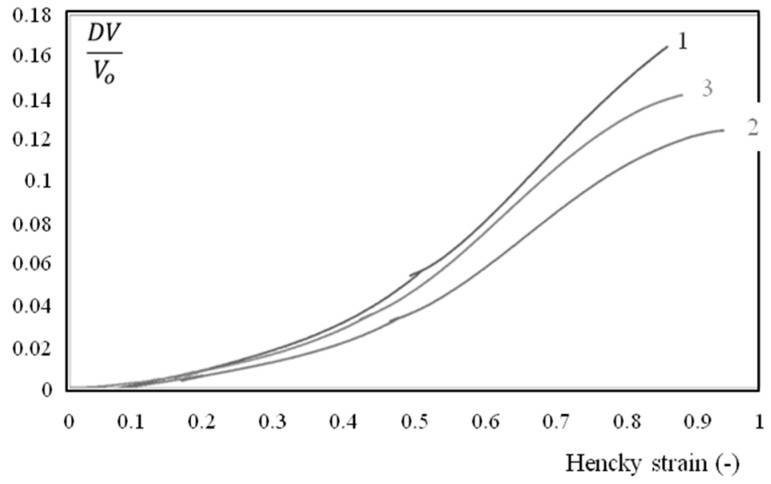
Volume deformation during a constant strain rate tensile test, for core (1), surface (2) and total thickness (3) specimens. The water content is 7% and samples were extracted from the first third of the plaque (close to the gate).

Nevertheless, the mechanical behavior varied significantly between surface and core samples (Figure 12 and Figure 13), even if the humidity core appeared more rigid and less isochoric.

## 4. Discussion and Conclusions

The first conclusion from our local measurements is that the mechanical behavior of injection-molded plaques cannot be uniform. This is not experimental scattering, but a consequence of the microstructure evolution that can be rather subtle. When samples are extracted from one part without taking note of the exact location, it may appear that the results are not reproducible (e.g., Figure 4) when in fact they are. This highlights the fact that the experimental protocol must carefully define the way samples need to be extracted. Such a remark is rather important when measurements deal with characterizing relationships between microstructure (or processing conditions) and mechanical properties.

The main mechanical parameters are not only the apparent moduli or yield. In our case, the main effect of the microstructure was on volume strain, suggesting that damage could be impacted in a significant manner. This highlights the fact that a one-dimensional (1D) standard mechanical analysis might not be advanced enough to address microstructure–properties relationships.

The change in microstructure can concern only lower levels of description. Consequently, usual macroscopic parameters (e.g., crystallinity ratio, skin thickness) may not reveal the lack of uniformity of the part. In other words, the crystallinity ratio, skin thickness and spherulite size are not enough to address the mechanical properties of the part. In our study, these parameters did not vary significantly while the behavior did vary.

Indeed, the lamellae scale was the relevant scale to be addressed to analyze the mechanical properties of the PA 66.

By the end of this first step, it seemed that the evolution of microstructure from the surface to the core and from the entrance to the bottom of the mold was related to an increase in amorphous mobility similar to that induced by humidity.

**Figure 14:**
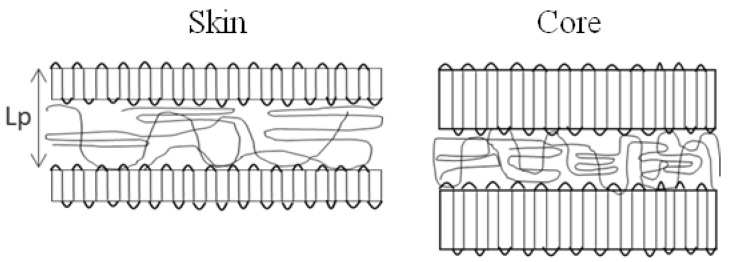
Possible lamellae organization in injection-molded PA66.

Our microstructural observations suggest that a gradient (at this level of description) exists from the extreme surface to the core and along the flow path. A possible schematic of the lamellae organization is given in Figure 14, as the lamellae are smaller and the amorphous phase is less constrained at the surface and further from the gate than that in the core and closer to the gate. This can explain the loss of mechanical rigidity and the change of incompressibility.

If this were confirmed, relevant microstructural parameters could be the state of the amorphous phase and the perfection of the crystal (or the mechanical contrast between the two), which would control the deformability of the “crystal/amorphous” stack. Obviously, one must also envisage link molecules that we did not address here. This is in qualitative agreement with recent observations including those deduced from in situ synchrotron analysis [26], but it must be confirmed.

This work has to be completed by exploring different processing conditions. It would also be interesting to promote a controlled microstructure (independent of injection molding) to validate the proposed schematic.

However, modeling could take advantage of such an analysis by guiding the choice of internal variables to be considered.

## Figures and Tables

**Table 1:** Melting temperature, *T_f_*, average lamellae thickness, <*L_c_*>, melting enthalpy, Δ*H_f_* and apparent crystallinity ratio in mass, X_c_, regarding surface and core samples.

Specimen	*T_f_* (°C)	<*L_c_*> (Å)	Δ*H_f_* (J/g)	X_c_ (%)
Surface	264.5 ± 0.3	238 ± 13	77.4 ± 1.4	39.5 ± 0.6
Core	266.0 ± 0.1	331 ± 11	74.3 ± 0.8	37.9 ± 0.3

**Table 2:** Microstructure indexes deduced from wide-angle X-ray scattering (WAXS) experiments.

Specimen	Crystallinity Index	Long Period (Å)	Crystalline Perfection	∆θ_am_	(100) Lamellae Thickness (Å)
Surface	0.43 ± 0.02	86	3.27 ± 0.05	8.08	74
Core	0.45 ± 0.02	97	3.91 ± 0.05	5.92	76
